# Cross talks between the immune and lymphatic endothelial cells regulate inflammatory lymph node lymphangiogenesis: defining a new therapeutic approach

**DOI:** 10.1186/2051-1426-1-S1-P184

**Published:** 2013-11-07

**Authors:** Sonia Elhadad

**Affiliations:** 1 Memorial Sloan Kettering Cancer Center, New York, NY, USA

## Introduction

The lymphatic system serves as the primary route for the metastasis of many cancers and the extent of lymphangiogenesis involvement is the most important indicator of tumor aggressiveness. Despite the apparent importance of the lymphatic vessels for tumor dissemination, it has remained unclear whether immune cells and lymphatic endothelial cells interactions may affect tumor progression and metastasis. Recent evidences indicate regulation of lymph node lymphangiogenesis by T lymphocytes. However, the nature of the T cells involved and the nature of the immune response in inflammatory lymph node lymphangiogenesis are still unveiled. We therefore characterized the nature of the inflammatory response in inflammatory lymph node lymphangiogenesis.

## Methods

We used a mouse model of inflammatory lymph node lymphangiogenesis and analyzed the nature of the immune response mounted during lymph node lymphangiogenesis and its role in regulating this pathophysiological process using a combination of flow cytometry, immunohistochemistry, cytokine analysis, and RT-PCR.

## Results

Inflammatory lymph node lymphangiogenesis is a multi step process defined by initiation, expansion and a return to quiescence. We found that each step is tightly orchestrated by a specific T cell response. At steady state lymph node lymphangiogenesis is negatively regulated by the anti lymphangiogenic cytokine IFNγ, produced by CD8α T cells. Initiation of inflammatory lymph node lymphangiogenesis results in suppression of CD8α producing IFNγ T cells and of Th17 cells, and in expansion of Th1 and Th2 cells. Furthermore, inflammatory lymph node lymphangiogenesis is increased in mice deficient in CD4 or CD8 T cells, suggesting a regulating role for each of the T cell subtypes. The effect of the T cell on inflammatory lymph node lymphangiogensis is exerted by regulating the production of prolymphangiogenic cytokines.

## Conclusion

Our data suggest that lymphangiogenesis can be targeted therapeutically at different stages, and by different way. This can be a novel strategy to control tumor spread.

